# Dispositional Mindfulness Co-Varies with Smaller Amygdala and Caudate Volumes in Community Adults

**DOI:** 10.1371/journal.pone.0064574

**Published:** 2013-05-22

**Authors:** Adrienne A. Taren, J. David Creswell, Peter J. Gianaros

**Affiliations:** 1 Department of Neuroscience, University of Pittsburgh, Pittsburgh, Pennsylvania, United States of America; 2 Department of Psychology, Carnegie Mellon University, Pittsburgh, Pennsylvania, United States of America; 3 Department of Psychology, University of Pittsburgh, Pittsburgh, Pennsylvania, United States of America; University of Medicine & Dentistry of NJ - New Jersey Medical School, United States of America

## Abstract

Mindfulness, a psychological process reflecting attention and awareness to what is happening in the present moment, has been associated with increased well-being and decreased depression and anxiety in both healthy and patient populations. However, little research has explored underlying neural pathways. Recent work suggests that mindfulness (and mindfulness training interventions) may foster neuroplastic changes in cortico-limbic circuits responsible for stress and emotion regulation. Building on this work, we hypothesized that higher levels of dispositional mindfulness would be associated with decreased grey matter volume in the amgydala. In the present study, a self-report measure of dispositional mindfulness and structural MRI images were obtained from 155 healthy community adults. Volumetric analyses showed that higher dispositional mindfulness is associated with decreased grey matter volume in the right amygdala, and exploratory analyses revealed that higher dispositional mindfulness is also associated with decreased grey matter volume in the left caudate. Moreover, secondary analyses indicate that these amygdala and caudate volume associations persist after controlling for relevant demographic and individual difference factors (i.e., age, total grey matter volume, neuroticism, depression). Such volumetric differences may help explain why mindful individuals have reduced stress reactivity, and suggest new candidate structural neurobiological pathways linking mindfulness with mental and physical health outcomes.

## Introduction

Mindfulness is a process involving attention and receptivity to what is happening in one's moment-by-moment experience [1], [2]. In addition to a growing mindfulness meditation training literature which focuses on fostering mindful awareness for health and well-being [3], a great deal of recent interest has also focused on the development of self-report state and trait mindfulness questionnaires which can measure naturally-occurring levels of mindfulness [4]. Although it is currently debated whether mindfulness meditation training and self-report questionnaire measures of mindfulness describe the same underlying mindfulness construct (e.g. [5]), recent studies indicate some correspondence, showing that short-term mindfulness meditation training programs increase dispositional self-reported mindfulness [5], [6], [7]. Notably mindfulness, as measured by self-report or (increased by) mindfulness-based interventions, is associated with improved well-being and decreased depression, anxiety, and chronic pain [8], [9], [10]. Additionally, mindfulness has been associated with positive health outcomes in a variety of stressed patient populations, including those with chronic pain, HIV, cancer, cardiovascular disease, and fibromyalgia [11], [12]. Despite the number of studies reporting positive associations between mindfulness and physical and mental well-being, it is still unclear how mindfulness produces these effects at the neurobiological level [13].

It is possible that mindfulness and mindfulness interventions improve health by reducing stress responding and concomitant stress-related physical health problems. For example, studies show that mindful individuals have reduced stress reactivity [14], [15], and patient studies indicate reduced stress-related symptomatology in rheumatoid arthritis [16], [17], inflammatory joint diseases [18], fibromyalgia [19], [20], and HIV [21]. Although little is known about the neurobiology of mindfulness [13], [22], neural regulation of the stress response appears to involve interaction between limbic regions and circuitry involved in reward and memory – including the amygdala and hippocampus—with the amygdala particularly implicated in gating stress responding [23], [24]. During acute stressors, the amygdala (and related subcortical structures) orchestrate the brain's rapid fight or flight response [23], which can be adaptive in some contexts but repeated, excessive, or prolonged stress responses (including amygdala reactivity) are thought to place organisms at risk for a broad range of stress-related diseases [25], [26], [27], [28]. Indeed, the amygdala has been shown to be a key player in mental and emotional health, with abnormal amygdala function identified in depression, anxiety, posttraumatic stress disorder, phobias, and panic disorders [27], [28], [29], [30], [31], [32], [33]. And moreover, some recent work suggests that reductions in perceived stress covary with reduced amygdala gray matter density [34]. The hippocampus also plays an important role in the neurobiology of stress: not only does it facilitate learning and memory [35], but it plays an important role in the regulation of stress responding via negative feedback regulation of the HPA-axis [24], [36].

Given the posited connections between mindfulness, neural stress responding, and health, the present study aims to test for relationships between dispositional mindfulness and limbic volumes - including amygdala and hippocampus – in a large community sample. Emerging functional and structural imaging studies highlight the potential for dispositional mindfulness (and mindfulness training) in altering the function and structure of these limbic regions [37], [38], [39], [40]. For example, mindful individuals have reduced resting state amygdala activity [40], and reduced amygdala activity when instructed to regulate their emotional response using affect labeling [37]. Structural changes in the hippocampus – namely, increased volumes – have also been observed in advanced mindfulness meditation practitioners [12], [41]. Several studies also report increased activation of the hippocampus or parahippocampal region during meditation [38], [39], [42]. Changes in grey matter density and cortical thickness have been reported in additional brain regions among regular mindfulness practitioners [12], [41], [42], [43], [44], [45], [46]. Increases in regional grey matter density have been observed in the left hippocampus, posterior cingulate cortex, temporo-parietal junction, and cerebellum after an 8-week Mindfulness-Based Stress Reduction (MBSR) training program [12]. Studies of experienced meditation practitioners have observed increased cortical thickness in the prefrontal cortex and right anterior insula compared to matched controls [46], [47], as well as greater grey matter concentration in right anterior insula, left inferior temporal gyrus, and the right hippocampus [41].

The present study provides the first test of whether dispositional mindfulness – using the Mindfulness Attention and Awareness Scale (MAAS) – co-varies with brain morphology in a community sample (N = 155). Building on the current body of research linking mindfulness to structural and functional brain changes, we tested several hypotheses about how dispositional mindfulness is associated with differences in brain tissue volume. Specifically, we hypothesized that higher levels of dispositional mindfulness would be associated with decreased grey matter volume in the amygdala and increased grey matter volume in hippocampus, based on previous mindfulness research implicating these subcortical structures in emotional reactivity and affect processing. While the current literature linking mindfulness to structural or functional brain changes did not offer any *a priori* hypotheses about other specific limbic or basal ganglia brain regions, we also conducted exploratory regression analyses relating dispositional mindfulness to bilateral caudate and nucleus accumbens, as these reward-related regions of the basal ganglia have been shown to be important for processing and responding to emotional stimuli [48], [49].

## Methods

### Participants

155 healthy adults (78 men, 77 women; mean age, 40.7±6.2 SD, range = 30–50 years) were recruited from the community by mass mailings to residents of Allegheny County, PA. The ethnicity of the sample was Caucasian/White (70.3%), African American/Black (21.9%), Asian (5.8%), and multiracial or other (1.9%). Inclusion criteria included no history of (1) cardiovascular disease (including treatment for or diagnoses of hypertension, stroke, myocardial infarction, congestive heart failure, and atrial or ventricular arrhythmias); (2) prior neurosurgery or neurological disorder; (3) current treatment for or self-reported psychiatric disorder; (4) typical consumption of greater than 15 alcoholic beverages per week; (5) daily use of corticosteroid inhaler; (6) current use of psychotropic, lipid lowering, or any cardiovascular medication, including any medication to control blood pressure; (7) metal implants or exposure; (8) colorblindness; and (9) claustrophobia. All participants were right-handed, as assessed by the Edinburgh Handedness Inventory [50]. Women were excluded if pregnant (verified by urine test). All participants gave written informed consent as part of protocols approved by the Institutional Review Boards of the University of Pittsburgh and Carnegie Mellon University. All analyses were based on a final sample size of 145 participants (10 participants were excluded during data analysis due to missing variables needed to compute regression analyses (n = 6) or missing/poor quality structural images (n = 4)). Informed consent was provided by all study volunteers and all study procedures were approved by the University of Pittsburgh and Carnegie Mellon University Institutional Review Boards.

### Procedure

The present study describes measures collected as part of the Pittsburgh Imaging Project (PIP), which has the aim of understanding the neurobiological, psychosocial, and behavioral correlates of health among community adults. For the present study, participants completed a psychosocial survey battery, which included the 15-item Mindful Attention Awareness Scale (MAAS). The MAAS assesses central characteristics of dispositional mindfulness, including attention to the present and awareness of everyday experiences (e.g. “I could be experiencing some emotion and not be conscious of it until some time later,” “I rush through activities without being really attentive to them”). Using a six-point Likert scale, subjects indicate how often they feel they experience these items on a day-to-day basis (“almost always” to “almost never,” all items were scored such that higher scores indicate higher mindfulness, sample α = .87). The MAAS has been previously validated using a variety of subject populations, including college students, cancer patients, and community adults [1], [51]. Previous studies of dispositional MAAS indicate an average mean score of 4.20 and 3.83 in samples of community adults (*n* = 436) and college students (*n* = 2277), respectively [1], [51], and mean MAAS score has been shown in some studies to significantly increase following MBSR training (from 3.88 to 4.69 [52]). As described below (see Statistical Analyses), psychosocial measures of neuroticism [53] and depressive symptomatology [54], [55] were collected and used as control variables in secondary analyses (Tables 1, 2).

**Table 1:** Bivariate correlations between MAAS and Psychosocial Affectivity Measures.

	R	*p*-value	n
BDI Total Score (0–63)	−0.168	0.037	154
PANAS: Positive Affect	0.250	0.002	153
PANAS: Negative Affect	−0.355	0.000	153
STAI Trait Anxiety	−0.328	0.000	154
NEO-N Neuroticism	−0.386	0.000	153
NEO-E Extraversion	0.252	0.002	152
NEO-C Conscientiousness	0.309	0.000	153

*Notes: MAAS = Mindfulness Attention Awareness Scale, BDI = Beck Depression Inventory, PANAS = Positive and Negative Affect Scale, STAI = State Trait Anxiety Inventory.*

**Table 2:** Subject Demographics (n = 155).

Variable	Mean	St. Dev.
Age	40.7	6.16
Gender	78 male, 77 female	-
Household Income	$38,519	$16,862
Years of School	17.12	3.24
MAAS: Mindfulness Score (1–6)	4.47	0.70
BDI Total Score	3.65	3.64
STAI: Trait Anxiety	33.19	7.66
PANAS: Positive Affect (1–5)	3.58	0.59
PANAS: Negative Affect (1–5)	1.61	0.52
NEO-N: Neuroticism	76.66	22.75
NEO-E: Extraversion	113.77	18.87
NEO-C: Conscientiousness	120.98	18.55

*Notes: MAAS = Mindfulness Attention Awareness Scale, BDI = Beck Depression Inventory, PANAS = Positive and Negative Affect Scale, STAI = State Trait Anxiety Inventory.*

Participants also completed a separate neuroimaging session. Images were acquired on a 3 Tesla Trio TIM whole-body scanner (Siemens, Erlangen, Germany), equipped with a 12-channel phased-array head coil. Three-dimensional magnetization prepared rapid gradient echo (MPRAGE) high-resolution T_1_-weighted neuroanatomical images were acquired for each subject over 7 minutes 17 seconds by these parameters: field of view = 256×208 mm, matrix size = 256×208 mm, time to repetition = 2100 ms, time-to-inversion = 1100 ms, time to echo = 3.29 ms, and flip angle = 8° (192 slices, 1 mm thick, no gap). MPRAGE images were used to derive volumetric measures described below.

### Structural Brain Image Analysis

For segmentation and volumetric analysis of the regions of interest (ROIs) in line with study hypotheses (i.e., amygdala, hippocampus, caudate nucleus, and nucleus accumbens), we used the Oxford University Centre for Functional MRI of the Brain (FMRIB) Integrated Registration and Segmentation Tool (FIRST) in the FMRIB Software Library (FSL) version 4.0. FIRST is a semi-automated model-based subcortical segmentation tool that relies on a Bayesian framework, as well as shape and appearance models obtained from manually segmented images provided by the Center for Morphometric Analysis, Massachusetts General Hospital (Boston, MA). Volumetric labels are parameterized by a three-dimensional deformation of a surface model based on multivariate Gaussian assumptions. Specifically, FIRST searches through linear combinations of shape modes of variation for the most probable shape given the intensity distribution in the T_1_-weighted image (for a more detailed description of this method, see [56]).

For volumetric processing, a two-stage affine registration to a standard space template (Montreal Neurological Institute space) with 1 mm resolution using 12 degrees of freedom and a subcortical mask was run to exclude voxels outside of subcortical regions. Second, the amygdala, hippocampus, caudate nucleus, putamen, nucleus accumbens, and globus pallidus were segmented with 50, 30, 30, 40, 50, and 40 modes of variation, respectively. Modes of variation were optimized based on a leave-one-out cross-validation using the training set [56]. Finally, boundary correction was implemented for each structure to classify boundary voxels as belonging to the structure or not using a statistical probability threshold (*z* score >3.00; *p*<0.001). The volume for each structure was then measured in mm^3^. Segmentations from each participant were visibly checked for any significant errors that could have occurred during the segmentation process (no errors were noted).

### Statistical Analyses

Summed total and regional grey matter volumes values were imported into Statistical Package for the Social Sciences (SPSS) 19.0 (IBM Corp. Released 2010. IBM SPSS Statistics for Windows, Version 19.0. Armonk, NY: IBM Corp.). In our first wave of analyses, we first tested for the strength of relationship between dispositional mindfulness and regional grey matter volumes (using Pearson's correlations). These bivariate correlations between MAAS score and grey matter volume were first assessed for significance (two-tailed, α<.05). We then conducted a second wave of analyses that controlled for individual difference variables implicated in volumetric effects using multiple regression analyses in SPSS. In order to conduct this secondary wave of analyses, we first created a MAAS variable that controlled for age, BDI, NEO-N, and total grey matter by regressing these person-level control variables onto the MAAS variable, and saved the standardized residuals. This residualized MAAS variable was then used in subsequent multiple regression analyses, testing whether residualized MAAS was associated with regional grey matter volumes in segmented regions.

## Results

### MAAS Associations with Grey Matter Volumes

**Figure 1:**
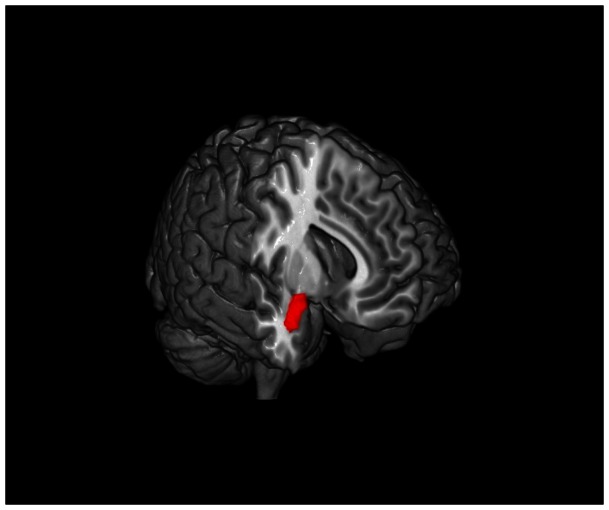
Greater dispositional mindfulness is associated with decreased grey matter volume in the right amygdala. The right amygdala is shown here in red.

We predicted that dispositional mindfulness would be negatively associated with amygdala volumes, and positively associated with hippocampal volumes. Consistent with our first prediction, we observed a significant negative association between dispositional mindfulness and regional gray matter volume in right amygdala (R = −0.203, *p* = 0.013) but not in the left amygdala (R = −0.095, *p* = 0.248) (Figure 1). Contrary to predictions, dispositional mindfulness was significantly negatively associated with regional gray matter volume in the right hippocampus (R = −0.201, *p* = 0.014) (but there was no association with left hippocampal volume) (Table 3).

**Table 3:** Multiple regression analysis relating dispositional mindfulness (Trait MAAS) and grey matter volumes.

Analysis	Correlation	*p*-value (two-tailed)	DF
MAAS & right amygdala volume	−0.175	0.035	143
MAAS & left amygdala volume	−0.017	0.838	143
MAAS & left caudate volume	−0.172	0.039	143
MAAS & right caudate volume	−0.114	0.174	143
MAAS & left nucleus accumbens volume	−0.076	0.361	143
MAAS & right nucleus accumbens volume	−0.041	0.629	143
MAAS & left hippocampus volume	−0.036	0.672	143
MAAS & right hippocampus volume	−0.073	0.381	143
Control variables:	Grey matter volume (by FSL), age (yrs), gender, BDI total score, NEO-N: Neuroticism		

*Notes: MAAS = Mindfulness Attention Awareness Scale, BDI = Beck Depression Inventory.*

**Figure 2:**
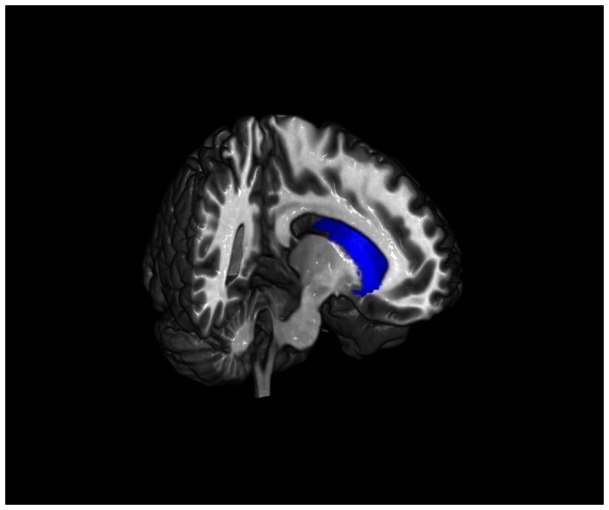
Greater dispositional mindfulness is associated with decreased grey matter volume in the left caudate. The left caudate is shown here in blue.

The extant literature offers no predictions about the relationship between mindfulness and regional gray matter volumes in caudate and nucleus accumbens. Nonetheless, we conducted exploratory analyses with these ROIs. These analyses revealed significant negative associations between MAAS score and regional gray matter volume in left caudate (R = −0.224, *p* = 0.006), right caudate (R = −0.194, *p* = 0.017), and left nucleus accumbens (R = −0.198, *p* = 0.015) (the association with right nucleus accumbens was not significant; R = −0.145, *p* = 0.078) (Table 3). Only the correlations between MAAS and left caudate remained significant after Bonferroni-correction for multiple tests (*α* = 0.00625) (Figure 2).

### Residualized MAAS Associations with Grey Matter Volumes

A more stringent secondary wave of analyses controlled for factors previously implicated in volumetric effects [57], [58], [59], [60], [61]. A significant negative relationship was observed between dispositional mindfulness and volume in the left caudate (*r* = −.172, *p* = 0.039,) and right amygdala (*r* = −.175, *p* = 0.035,) after controlling for subjects' age, gender, total grey matter volume, depressive symptomatology, and neuroticism (Table 3). Specifically, the relationship between more mindful individuals and smaller right amygdala and left caudate volumes persists even after controlling for person level factors previously shown to affect brain volumes.

## Discussion

The present study is the first study to examine the relationship between dispositional mindfulness and regional grey matter volume in a large sample of community adults. Although previous studies have shown that mindful individuals have altered amygdala responses (and connectivity) to affective stimuli [37], [40], [62], [63], [64], these studies have not tested for underlying structural differences in amygdala volumes. Notably, this study indicates that mindful individuals have smaller right amygdala volumes (an effect that survived controls for psychosocial and demographic factors), suggesting one potential neurobiological pathway for these functional amgydala reactivity effects. Similarly, amygdala reactivity is considered to be an important region for gating central stress responses [23]; thus smaller amygdala volumes may reflect a potential neurobiological mechanism for reduced stress reactivity in more mindful individuals [14], [15], [65], and lower negative affect in daily life [1]. The lack of an association between left amygdala volume and mindfulness may be attributable to functional hemispheric differences in affective processing; it has been previously suggested that the right amygdala may be primarily responsible for the immediate aggregate response to affective inputs and processing of affective visual stimuli, whereas the left amygdala is more finely attuned to detail and affect-related language [66], [67]. Furthermore, our findings are consistent with previous studies showing that mindfulness training effects are more robust for right amygdala (while minimally impacting functional activity in the left amygdala) [34], [37].

Contrary to predictions, dispositional mindfulness was not associated with increased hippocampal volumes. In fact, there was some weak evidence that dispositional mindfulness was associated with smaller right hippocampal volumes, although this association did not survive controls for psychosocial and demographic variables. This lack of association between mindfulness and hippocampal volumes was unexpected, given that previous structural studies have reported *increased* grey matter density in the hippocampus among regular mindfulness practitioners [41] and after MBSR training [12]. One potential explanation for this finding (although speculative and to be tested in future research) is that the hippocampus may differentiate individual difference measures of mindfulness from mindfulness meditation training effects. Specifically, mindfulness meditation requires one to actively acknowledge or notice their experience moment-by-moment, a process of consciously “remembering” your experience. The hippocampus is a structure critical for learning and memory [35], and it may be that mindfulness meditation practice activates hippocampus when one remembers or consciously acknowledges their experience. By contrast, dispositional measures of mindfulness, such as the MAAS, may reflect a more general capacity to control one's attention moment-by-moment [5], [41], thus relying more on attention regulation neural networks [68], [69] and less on hippocampal involvement. Indeed, previous studies are consistent with this possible explanation: the process of meditation has been associated with activation of hippocampus and parahippocampus [38], [39], [42]; by contrast, previous functional neuroimaging studies of dispositional mindfulness have not been associated with activation of hippocampus when participants are asked to attend to affective dimensions of their present-moment experience [37], [40], [64].

The present study provided one of the first opportunities to test whether dispositional mindfulness is associated with caudate volumes, and we provide preliminary evidence that more mindful individuals have smaller left and right caudate volumes (only the left caudate association survived Bonferroni correction for multiple comparisons). As part of the basal ganglia, the caudate's role in the reward response has been well established, but recent fMRI work has also implicated caudate in processing negative affect [70], [71], [72] and the neural response to sadness [73]. The present findings may thus suggest that reward responding as well as stress responding is altered in more mindful individuals, consistent with the enhanced affective regulation associated with mindfulness. Indeed, anecdotal reports from mindfulness meditation practitioners describe an increase in more quiescent mood states (e.g., serenity, calm) after mindfulness training [74]. Further, some recent meditation research implicates the caudate in meditation training effects. Lee et al. (2012) [75] note that during loving-kindness meditation, novices show decreased activation in right caudate at baseline compared to experts while viewing sad pictures. Although speculative, decreased caudate recruitment in response to negative emotional experiences in more mindful individuals could represent one possible mechanism linking lower caudate volume to greater mindfulness. We consider our initial findings between dispositional mindfulness and smaller caudate volumes to be promising new direction in developing a basic neurobiological models of mindfulness, but more research is needed.

The present study contributes to an emerging body of research relating individual differences to regional gray matter volumes. Previous research examining the relationship between personality traits and brain volumes has shown that individual differences in a variety of personality measures – including novelty seeking, harm avoidance, reward dependence, and persistence [76], extraversion, neuroticism, agreeableness, conscientiousness, and openness [77] - may reflect differences in the structural properties of different brain regions. In particular, individual differences in trait neuroticism have been negatively associated with the brain to intracranial volume ratio [60] and gray matter concentration in the right amygdala [78], and positively associated with gray matter volume in cingulate and left caudate [77]. These results are of particular interest in relation to our findings, as neuroticism has been used in these studies as an index of stress reactivity, particularly the anxiety-related subscales of neuroticism [60]. While increased stress reactivity is associated with decreased gray matter concentration in right amygdala and increased gray matter volume in left caudate in previous studies [77], [78], the present findings associate increased trait mindfulness with decreased gray matter volume in both right amygdala and left caudate (moreover, the mindfulness effect holds after controlling for any effect of neuroticism on volumes). Thus, mindfulness (and by extension, mindfulness meditation training) may be protective against the structural neural changes associated with negative affective traits.

### Limitations and Future Research Directions

Our ability to make causal inferences about the relationship between mindfulness and brain morphology is limited by the cross-sectional correlational design of the present study. Additionally, we look only at dispositional mindfulness, not the effects of mindfulness meditation training. Based on previous fMRI studies of mindfulness meditation, it may be the case that active mindfulness training engages additional brain regions in which volumetric effects would be seen (e.g. prefrontal regions); our analyses (using FSL-extracted limbic and basal ganglia volumes) did not allow us to look at cortex and the potential effects of increased prefrontal volumes and their purported regulatory effects (which is an important direction for future research). One assumption we have made in framing this work is that structural differences in brain volumes underlies differences in functional activation of these regions - a positive relationship between regional activation and volume has previously been shown using functional imaging [79], [80]; but much more research is needed in understanding function-structure relationships in neuroimaging studies.

The present study has several notable strengths for advancing our neurobiological understanding of mindfulness. This study is the largest mindfulness neuroimaging study to date, and we conducted rigorous analyses controlling for variables implicated in volumetric effects (e.g., depression, neuroticism, age) [57], [58], [59], [60], [61]. This study provides an initial indication that higher dispositional mindfulness is associated with decreased grey matter volume in the amygdala and caudate; these volumetric differences may help explain reduced stress reactivity in more mindful individuals.

### Conclusions

The present findings represent an important contribution to the current understanding of how mindfulness may reduce stress responding and thus improve physical and psychological health. Smaller grey matter volumes in subcortical structures, particularly the amygdala and caudate, may be the morphological correlates of the previously-reported link between trait mindfulness and reduced stress reactivity and improved well-being. These findings help identify candidate structural neurobiological pathways linking mindfulness with reduced stress and negative affectivity in a broad range of studies [3], [11].
